# Systemic methotrexate (MTX) in early pregnancy: a retrospective study of a tertiary maternity hospital

**DOI:** 10.1007/s11845-024-03748-9

**Published:** 2024-07-09

**Authors:** Ahmed Lutfi, Deirdre Hayes-Ryan, Elmarie Cottrell, Richard A. Greene

**Affiliations:** 1 Department of Obstetrics and Gynaecology, Cork University Maternity Hospital, Cork, Ireland; 2 Pregnancy Loss Research Group, Department of Obstetrics and Gynaecology, University College Cork, Cork, Ireland; 3 Department of Obstetrics and Gynaecology, National Perinatal Epidemiology Centre, University College Cork, Cork, Ireland; 4 Department of Pharmacy, Cork University Hospital, Cork, Ireland

**Keywords:** Early pregnancy, Ectopic pregnancy, Methotrexate, Pregnancy of unknown location

## Abstract

**Background:**

Methotrexate (MTX) is used in clinical practice as a medical treatment option in patients with early pregnancy complications like ectopic pregnancy.

**Aims:**

To review systemic MTX therapy use in the first trimester of pregnancy in our hospital and to examine subsequent clinical outcomes.

**Methods:**

Retrospective review of all women treated with systemic MTX in early pregnancy identified from electronic prescription records from 1 January 2018 to 31 December 2020 at Cork University Maternity Hospital, Ireland. Relevant data was transcribed from electronic health records.

**Results:**

Indications for treatment were tubal ectopic pregnancy (70%, *n* = 51), persistent pregnancy of unknown location (22%, *n* = 16) and caesarean scar pregnancy (7%, *n* = 5). Treatment was successful in 88% (*n* = 44) of tubal ectopic pregnancies with 73% (*n* = 37) and 14% (*n* = 7) of women receiving a single dose and repeated doses, respectively. Only 8% (*n* = 4) of tubal ectopic pregnancies required emergency surgery for subsequent tubal rupture. In 93% (*n* = 15) of cases of persistent pregnancy of unknown location, treatment was successful with one patient requiring uterine evacuation. Women with caesarean scar pregnancy were treated with combined MTX and uterine evacuation without complication.

**Conclusions:**

The efficacy of medical treatment with systemic MTX for confirmed tubal ectopic pregnancy in our hospital is in line with national and international standards. Careful consideration should be given to treating caesarean scar pregnancy and persistent pregnancy of unknown location with systemic MTX. Systemic MTX use guided by clinicians specialised in early pregnancy complications and safe medication practices may improve treatment success and reduce adverse events.

## Introduction

Ectopic pregnancy (EP) is defined as a pregnancy that implants outside of the uterine cavity with the majority (96%) implanting in a fallopian tube [1]. Women with symptomatic EP may present with vaginal bleeding, lower abdominal pain, diarrhoea and/or dizziness in the first trimester [2]. In the Republic of Ireland, the rate of hospitalisation from EP increased from 12.8/1000 deliveries in 2005 to 17.7/1000 deliveries in 2016 [3]. Severe morbidity associated with EP occurs in 4.8% of women due to complications such as intra-abdominal haemorrhage needing surgical intervention and blood transfusion [4]. Furthermore, EP contributed to 50% of direct maternal deaths in the first trimester of pregnancy in Ireland between the years 2009 and 2018 [4].

Historically, EPs were diagnosed through surgical intervention [5]; however, advances in early pregnancy knowledge and the use of transvaginal ultrasound have helped identify EP at earlier gestations [5]. Diagnosing EP can be challenging, as most EP appear as an inhomogeneous mass on sonography [6]. An extrauterine gestational sac may be present in 20–40% of cases, while an extrauterine sac with a yolk sac or foetal pole may represent 15–20% of cases [6]. Women with EP can also initially present as a pregnancy of unknown location (PUL), an interim diagnosis where sonography does not definitively identify a pregnancy’s location [7]. While the majority of women with PUL are subsequently diagnosed with a spontaneous miscarriage or viable intrauterine pregnancy, 7 to 20% will be reclassified as an EP [6, 7]. A minority of women with PUL are reclassified as persistent pregnancy of unknown location (PPUL) based on plateauing serum β-human chorionic gonadotrophin (β-hCG) levels [7]. Caesarean scar pregnancy (CSP) is a rare form of EP with incidence rates ranging from 1/1800 to 1/2500 of all pregnancies [6, 8]. With CSP, the gestational sac implants either fully or partially within a scar caused by a prior caesarean section [6, 8].

Earlier diagnoses of EP, prior to rupture and haemodynamic instability, allow for the option of medical treatment with methotrexate (MTX) [9]. MTX is a folic acid antagonist which inhibits DNA synthesis in actively proliferating cells like foetal cells [10]. Treatment with systemic MTX has been shown to be cost-effective in the management of EP when compared against surgical options [11]. In Ireland, MTX is given as a 50-mg/m^2^ intramuscular injection to treat EP provided that eligibility criteria according to national and local guidelines are satisfied [12]. There is no consensus regarding systemic MTX use for PPUL or CSP [8, 13]. The aim of this study was to examine the use of systemic MTX therapy in early pregnancy in our hospital and compare our inclusion criteria, success and morbidity to national and international standards [6, 12].

## Materials and methods

### Design and setting

This was a retrospective study of women who received systemic MTX therapy in the first trimester of pregnancy at Cork University Maternity Hospital (CUMH) from 1 January 2018 to 31 December 2020 inclusive (3-year period). CUMH is a tertiary-level maternity hospital with almost 8000 deliveries annually and a dedicated early pregnancy clinic (EPC) [13]. The EPC operates Monday to Friday and serves up to 5000 women annually [14]. In our hospital, patients undergo an informed consent process and are provided with verbal and written information on intramuscular MTX for EP. Women receiving systemic MTX are admitted to the gynaecology ward and have baseline blood tests performed to ensure normal renal, liver and haematological parameters. The women’s body surface area (BSA) is calculated for MTX dosing. MTX is given by intermuscular injection by clinical ward staff, and the patient is retained for a short period to ensure no adverse events. The patient is then scheduled to return to our EPC on day 4 and day 7 for serum β-hCG sampling and is given advice on symptoms of concern.

Eligible women were identified from the electronic pharmacy records of intramuscular MTX prescriptions using the Maternal & Newborn Clinical Management System. Electronic healthcare records were reviewed between April and November 2021. Ethical approval for the study was granted by the Clinical Research Ethics Committee of the Cork Teaching Hospitals (Ref: ECM 4 (q) 11/5/2021).

### Data collection

Demographic data was collected for all women including age and the presence of any risk factors for EP. Data pertaining to the decision for systemic MTX were recorded including indication for treatment, location at time of decision, ultrasound findings and serum β-hCG results (IU/L). The duration of surveillance prior to the decision and administration of systemic MTX was recorded. The number of days until discharge from follow-up was recorded. Information on the time of administration was collected. Outcomes following treatment were recorded including successful treatment with a single dose, requirement for repeat systemic MTX administration, requirement for surgical intervention and lack of adherence to follow-up. Any non-surgical adverse events after treatment were documented.

### Statistical analysis

Data was recorded using Microsoft Excel 2019 (Microsoft Corporation, Redmond, WA, USA) and was stored in accordance with the General Data Protection Regulation. Descriptive statistics were performed using SPSS 24 (IBM Corporation, Armonk, NY, USA) in December 2021. The Sharipo-Wilk test was used to evaluate the normal distribution of the data. Parametric data were presented as means and standard deviations, while non-parametric data were presented as median and range values.

## Results

### Demographics

During the 36-month time period reviewed, a total of 72 women received systemic MTX in early pregnancy in our hospital. The indications for treatment were tubal EP (71%, *n* = 51), caesarean scar pregnancies (CSP; 7%, *n* = 5) and PPUL (22%, *n* = 16). Patient demographics at the time of administration of systemic MTX are presented in Table 1. All women were haemodynamically stable with no documented medical contraindications to treatment; however, five women (7%) did not have serum liver/renal function tests performed prior to administration. All decisions for treatment were made by senior clinicians (senior/specialist registrar or consultant) with decisions for treatment being made half the time in the EPC (51%, *n* = 37) and the remainder of the time following admission to the hospital with early pregnancy complications (48%, *n* = 35). Systemic MTX was administered within weekday daytime hours (0800–1800) in 57 women (79%) with the remainder receiving treatment outside this time and on weekends.

**Table 1:** Patient characteristics, results of investigations and other service features according to treatment diagnosis and prior to methotrexate administration (n = 72)

**Patient characteristics**	**Treatment diagnosis**		
	**Tubal EP** ***n* = 51**	**CSP** ***n* = 5**	**PPUL** ***n* = 16**
Age (years)	33 (23–44)	34 (22–43)	30 (25–40)
Risk factors for EP present	49% (*n* = 25)	100% (*n* = 5)	44% (*n* = 7)
Haemodynamically stable	100% (*n* = 51)	100% (*n* = 5)	100% (*n* = 16)
No medical contraindication to MTX	100% (*n* = 51)	100% (*n* = 5)	100% (*n* = 16)
Full blood count, liver/renal profile measured	92% (*n* = 47)	100% (*n* = 5)	100% (*n* = 16)
**Ultrasound findings**			
EP mass diameter (mm)	14.2 (4.9–36.3)	14.3 (7.3–21.8)	-
Fetal cardiac activity present	2% (*n* = 1)	40% (*n* = 2)	-
Free fluid in pelvis	33% (*n* = 17)	0% (*n* = 0)	38% (*n* = 6)
**Serum β-hCG results**			
Initial levels (U/L)	678 (62–5231)	10,579 (2547–26,284)	529 (24–9265)
Number of serum β-HCG samples collected	3 (1–6)	2 (0–8)	4 (2–8)
Levels at decision for MTX (U/L)	1134 (55–5272)	12,821 (2626–26,284)	1009 (28–9606)
**Other service findings**			
Decision by senior clinician/consultant	100% (*n* = 51)	100% (*n* = 5)	100% (*n* = 16)
Duration of surveillance (days)	7 (1–21)	15 (5–18)	8 (3–28)
Decision made in EPC	49% (*n* = 25)	40% (*n* = 2)	63% (*n* = 10)
MTX given within weekday working hours (0800–1800)	78% (*n* = 40)	100% (*n* = 5)	75% (*n* = 12)

Data presented as percentage (number) or median (range)

*β-hCG* β-human chorionic gonadotropin, *CSP* caesarean scar ectopic pregnancy, *EPC* early pregnancy clinic, *EP* ectopic pregnancy, *MTX* methotrexate, *PPUL* persistent pregnancy of unknown location

### Tubal ectopic pregnancies (EP)

**Fig. 1:**
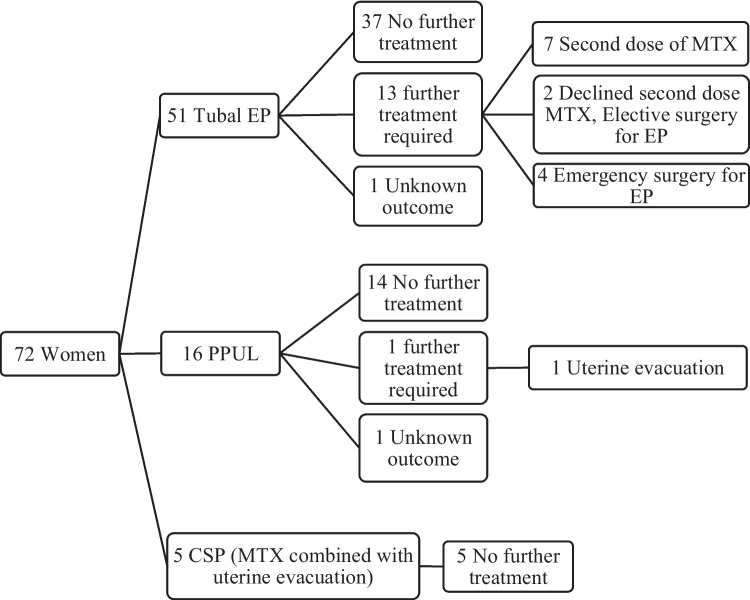
Patient outcomes following methotrexate treatment in early pregnancy CSP, caesarean scar pregnancy; EP, ectopic pregnancy; MTX, methotrexate; PPUL, persistent pregnancy of unknown location.

The median tubal EP mass size was 14.2 mm (range 4.9–36.3 mm) with only one patient having a mass size > 35mm who was treated successfully with one dose of systemic MTX. Foetal cardiac activity was present in one patient (2%) who had a presenting serum β-hCG of 1771 IU/L and mass size of 10.8 mm and was treated successfully with two doses of systemic MTX. There was a wide range in the levels of serum β-hCG prior to systemic MTX administration with 17 women having a serum β-hCG level > 1500 IU/L and one patient having a serum β-hCG level > 5000 IU/L. The serum β-hCG of 73% (*n* = 37) of women returned to non-pregnant values following treatment with one dose, while 25% (*n* = 13) of women required further treatment (Fig. 1). Of those requiring further treatment, 14% (*n* = 7) opted to receive a second dose of systemic MTX while 4% (*n* = 2) opted for elective surgery. Emergency surgery for suspected tubal EP rupture was necessary for 8% (*n* = 4) of women, occurring between 5 and 24 days subsequent to treatment (median 11.5 days). All women requiring emergency surgery for suspected tubal rupture (*n* = 4) had serum β-hCG levels > 1500 IU/L at the time of administration of MTX. The initial MTX treatment was given within working hours for the majority of patients who needed emergency surgery for EP (75%, *n* = 3) and in the majority of patients who needed a repeat MTX (87%, *n* = 6). One patient with tubal EP was not compliant with follow-up and their outcome is unknown. One patient with tubal EP inadvertently received MTX intravenously (outside of working hours) and subsequently suffered an upper limb thromboembolic event. The median number of days of follow-up after initial MTX treatment was 24 days (range 14–58).

### Persistent pregnancy of unknown location (PPUL)

Women diagnosed with PPUL had a median of two (range 1–4) transvaginal ultrasound scans prior to receiving systemic MTX. The median number of serum β-hCG samples collected before receiving treatment was four (range 2–8) while the median number of days of surveillance prior to treatment was eight (range 3–28). The median serum β-hCG level at decision for treatment was 1009 IU/L (range 28–9606 IU/L). The serum β-hCG of 88% (*n* = 14) of women returned to non-pregnant values. One patient with PPUL needed an emergency uterine evacuation procedure to manage abdominal pain and heavy vaginal bleeding due to a non-viable intrauterine pregnancy identified subsequent to treatment. One patient with PPUL was not compliant with follow up and their outcome is unknown. The median number of days until discharge after initial MTX treatment was 28.5 days (range 13–41).

### Caesarean scar pregnancies (CSP)

As part of treatment for CSP, five women received systemic MTX prior to proceeding with the elective uterine evacuation procedure on day 1 (80%, *n* = 4) and day 2 (20%, *n* = 1) after treatment. Different staff performed the uterine evacuations, and the procedures involved both suction and manual curettage. The median CSP mass size was 14.3 mm (range 7.3–21.8 mm) with foetal cardiac activity present in two cases. The median serum β-hCG level at decision for MTX in women with CSP was 12,821 IU/L (range 2626–26,284 IU/L). No further intervention was required for these women following combined treatment with MTX and the uterine evacuation procedure. Women receiving treatment for CSP were followed up for a median of 23 days (21–50).

## Discussion

In this 36-month retrospective study, systemic MTX was administered to women diagnosed with tubal EP, CSP and PPUL in early pregnancy. Though the majority of women were successfully treated, we identified that the use of systemic MTX differs among clinicians based on the results of sonography, blood tests and period of surveillance.

We report a success rate of 88% in women with tubal EP treated with systemic MTX (single dose and repeated doses) in keeping with success rates in other studies, which range from 65 to 95% [15–17]. The inclusion and exclusion criteria for systemic MTX in treating tubal EP varied in our study in keeping with the literature [18–20]. Most authors agree on the use of MTX preferentially in cases of hemodynamic stability and absence of foetal cardiac activity [6, 12, 18–20]. The presence of foetal cardiac activity in tubal EP is a contraindication to MTX treatment due to high rates of failure and morbidity. The woman in our study with foetal cardiac activity who opted for MTX treatment did so following extensive counselling having declined elective surgery. There are adverse effects associated with MTX such as marrow suppression, renal injury and liver injury; therefore, studies emphasise the importance of excluding renal, liver or haematologic diseases prior to MTX treatment (which is an area for improvement in our hospital) [6, 12, 18–20].

Pre-MTX serum β-hCG cut-off values for tubal EP differ in the literature and in our study [21–23]. According to Irish guidelines on tubal EP, systemic MTX should be considered when serum β-hCG levels are < 1500 IU/L [12]. Though pre-MTX serum β-hCG levels up to 5000 IU/L have been described in women with tubal EP, the risk of emergency surgery rises with increasing serum β-hCG levels [21, 22, 24]. Thresholds using the size of tubal EP also vary with no consensus on whether size is predictive of successful outcomes [23, 25]. Irish guidelines on tubal EP suggest using systemic MTX when the size is < 35 mm [12]. Only one patient in our study received treatment with tubal EP size measurements larger than recommended by national standards, but this patient was treated successfully. Outcomes following treatment for our women with tubal EP were similar to Irish standards, which report an incidence rate of 15% for repeat MTX administration and approximately 7% for emergency surgery [12].

Case reviews of women with CSP describe success rates of up to 41% with systemic MTX alone, and to improve success, treatment may be paired with evacuation or excision of the pregnancy [8]. Planned surgery has been described up to 7 days after systemic MTX in women with CSP [8]. Current national guidelines do not provide further details on the factors that influence CSP management decisions like CSP imaging features (e.g. size, viability, myometrial thickness) [12]. Newer studies on CSP management comparing combined MTX and uterine evacuation with uterine evacuation alone have shown that preoperative MTX did not reduce intraoperative estimated blood loss [26]. There was no significant difference in the presence of retained products of conception on follow-up scans treated with combined treatment and uterine evacuation alone [26]. This suggests that preoperative MTX does not provide additional benefit to women with CSP. A recent systematic review and network meta-analysis which assessed a total of 8369 women and 17 different CSP treatment modalities concluded that the use of MTX (both locally injected or systemically given) as a standalone treatment is not recommended [27]. This analysis which included 73 trials (with seven randomised controlled trials) identified that operative options such as laparoscopy, transvaginal resection, hysteroscopic curettage and high-intensity focused ultrasound combined with suction curettage were the most efficacious treatment options for CSP and exhibited the least complications [27]. Although the primary focus of this review was not on CSP, it is worth noting that due to its current relevance, our unit’s success with MTX in these cases merits further examination and suggests the need for additional review. Our CSP cases were treated successfully, but perhaps they would have benefited from operative management and avoided systemic MTX and its potential side effects.

There is no national or universal definition for PPUL and no recommendations for when to use systemic MTX in PPUL. This is reflected in our study by the differences in the number of scans performed, the number of serum β-hCG levels collected, the duration of surveillance and serum β-hCG treatment thresholds prior to treatment in women diagnosed with PPUL. PPUL diagnoses recorded in this study were according to the impression of the responsible clinician. Though our success rate with MTX therapy for women with PPUL was 93%, the use of systemic MTX for women with PPUL is controversial. There is a risk of misdiagnosing an intrauterine pregnancy as a PPUL as demonstrated in our study. MTX-induced embryopathy is a serious and avoidable complication occurring when a viable pregnancy is misdiagnosed as an EP or PPUL and is exposed to MTX [28].

Many early pregnancy specialists advocate that systemic MTX should never be given empirically in cases of PUL as it is an interim diagnosis, but in PPUL, there is no consensus on the duration of surveillance or serum β-hCG threshold before offering MTX [13]. Clinical practice guidelines in France suggest that asymptomatic women with PPUL who are observed for at least 10 days and have serum β-hCG levels > 2000 (IU/L) may be offered MTX [29]. Women in our study with a diagnosis of PPUL had a large range of serum β-hCG levels before receiving MTX. A multicentre randomised control trial in the United States studying the optimal management strategy for PPUL found that active management of PPUL (MTX therapy alone or uterine evacuation followed by MTX) was more effective in achieving pregnancy resolution than expectant management [30, 31]. Furthermore, active management of PPUL resulted in less unscheduled surgical interventions than expectant management. However, the study did identify that women had a stronger preference for expectant management of PPUL by surveillance of serum β-hCG kinetics [30]. There is a need for further studies to better distinguish PUL from PPUL and to identify predictors for treatment success in women with PPUL given significant adverse side effects of MTX.

Given the varied MTX prescribing and administration practices in our unit, care standardisation would make the results of future studies on the use of MTX in early pregnancy more robust. In response to the findings of this review, we have updated our local MTX prescribing and administration guidelines. Changes include the development of an improved electronic prescription which will minimise human error dose calculations and provide enhanced clinical decision support for prescribers. We introduced dose-banding and the use of pre-filled MTX syringes to avoid cytotoxic spillage and waste. We advocate that systemic MTX be given by two healthcare staff to ensure correct administration and handling of biochemicals, and that timing of administration should be limited to weekday daytime hours when pharmacy and a full staff complement are present. To improve patient selection for treatment, this study proposes reserving decisions for systemic MTX to clinicians specialised in early pregnancy. Early pregnancy specialists can ensure that appropriate investigations have been performed and provide continuity of care. Shared decision-making for treatment could involve pharmacists, nurse specialists, sonographers, bereavement services and the patient. A multi-disciplinary approach to EPs and PUL may improve systemic MTX success rates and patient satisfaction.

### Strengths and limitations

This retrospective study was conducted in a single tertiary centre and, to the best of our knowledge, is the largest study in Ireland reviewing MTX use in early pregnancy. The findings have facilitated improvements in safe MTX prescribing and administration practices for women with early pregnancy complications. We recognise that a limitation of our study is that choice for systemic MTX may have been influenced by clinical presentations not documented in the women’s records. A second limitation is that the diagnosis of PPUL in this study was based on the assessments made by the attending clinician and that the thresholds or criteria for MTX treatment for PPUL were not clearly specified. Another limitation is that two women did not complete follow-up and their outcomes are unknown.

## Conclusions

Systemic MTX therapy in early pregnancy is effective in treating suitable candidates with EP and success rates in our study are similar to international figures. This study brings additional information regarding treating CSP and PPUL with MTX. There is scope for improvement in both patient selection and administration procedures for systemic MTX treatment in our hospital with local policy and protocol changes underway in order to facilitate this.
